# How loneliness increased among different age groups during COVID-19: a longitudinal analysis

**DOI:** 10.1007/s10433-023-00798-3

**Published:** 2024-01-03

**Authors:** Fiona Köster, Oliver Lipps

**Affiliations:** 1 LIVES, Institute of Social Sciences, University of Lausanne, Lausanne, Switzerland; 2 Swiss Centre of Expertise in the Social Sciences (FORS), Lausanne, Switzerland; 3 Institute of Sociology, University of Bern, Bern, Switzerland

**Keywords:** Loneliness, Age, Life course, Longitudinal, COVID-19, Pandemic

## Abstract

The COVID-19 pandemic entailed restrictions that hampered face-to-face interactions and social gatherings. In this paper, we examine whether loneliness increased to different extents among age groups due to these restrictions, and if these differences were mediated by specific life course conditions. Based on longitudinal data from the Swiss Household Panel, our results show that loneliness increased disproportionately among younger individuals during the pandemic. This finding aligns with the social convoy model and the socioemotional selectivity theory, which postulate a decline of social network size over the life course. It also corresponds to findings indicating a decrease in contact frequency with increasing age. Individuals aged 30 years and above experienced a lower increase in loneliness when they lived in shared households; however, this protective effect was not observed for younger individuals. Living together with a partner, being male, and not anticipating health complications in case of a COVID-19 infection moderated the increases of loneliness, but they were independent of age.

## Introduction

Loneliness has been recognized as a growing public health issue, that impacts both mental and physical health and is associated with an increased risk of morbidity and mortality (Hawkley and Cacioppo 2010; Holt-Lunstad et al. 2015). The occurrence and severity of loneliness has been aggravated by the COVID-19 pandemic due to restrictive measures that severely limited face-to-face contact.

These measures induced a substantial limitation of quantitative aspects of social relationships since the opportunities to interact in public were drastically reduced. In addition, the number of people with whom one was allowed to meet in private was severely limited. As social networks of younger people are generally larger (Wrzus et al. 2013) and quantitative aspects of social relationships more important for preventing the prevalence of loneliness (Victor and Yang 2012), we hypothesize that age-normative expectations should lead to a stronger increase in loneliness among adolescents and young adults during the COVID-19 pandemic than among middle-aged or older individuals. This assumption is based on the social convoy model and the socioemotional selectivity theory, which postulate a decline of social network size and decrease of contact frequency with age (Carstensen 1992; Kahn and Antonucci 1980). Furthermore, age is associated with different life course stages, which are likely to mediate loneliness, such as living arrangements or health (Luhmann and Hawkley 2016; Savikko et al. 2005). For instance, individuals aged 30 years and above generally have the means to voluntarily choose the household composition that they want to live in, whereas adolescents and young adults tend to lack the autonomy to make such a decision.

The COVID-19 pandemic allows us to analyze the evolution of loneliness caused by an exogenous event. While many articles rely on cross-sectional or retrospective data, we use a longitudinal design that measures individual changes of loneliness before and during the imposition of restrictions for a large, nationally representative sample. What the reduction of face-to-face interaction means for levels of loneliness of different age groups is still largely unexplored.

This paper is organized as follows: first, we define loneliness, before we depict age and life course-related differences regarding the prevalence of loneliness. We then formulate our expectations regarding the effects of COVID-19 restrictions on changes in loneliness between age groups. Thereafter, we present our data and show bivariate and multivariate results of changed loneliness levels due to COVID-19 restrictions. The findings and policy implications are discussed in the conclusion.

## Theoretical framework

Loneliness is a multidimensional phenomenon that arises when individuals perceive a discrepancy between the desired and actual quality and quantity of their social relationships (Perlman and Peplau 1981). While the quantitative aspect revolves around the frequency of social interactions and social network size, the qualitative aspect depends on the existence of meaningful social relationships and the feeling of closeness.

Resulting from the implementation of COVID-19 restrictions, the opportunities to satisfy the quantitative aspect of social relationships became notably impeded in many countries. Social interactions in the setting of public, work, or educational life were drastically reduced, and the number of individuals that were permitted to gather in person was equally limited. Apart from the unprecedented impact that restrictions had on social encounters, a deterioration of the quality of social relationships cannot be ruled out either as both aspects are interrelated and the absence of personal interactions and close personal contact might negatively influence qualitative aspects of relationships as well, thus possibly leading to increased loneliness.

### Loneliness by age

The occurrence of loneliness does not follow a linear trend over the life course. Although research tended to focus on the prevalence of loneliness among older adults, a growing number of studies has started to emphasize the impact and occurrence of loneliness among young and middle-aged adults. Previous findings indicate that loneliness is widespread during adolescence and young adulthood, lower around middle adulthood with a slight peak around late middle adulthood and is most pronounced at advanced old age, meaning 80 years and above (Dahlberg et al 2015; Jylhä 2004; Hawkley et al. 2022; Luhmann and Hawkley 2016). However, most findings regarding age-related differences are based on cross-sectional data (Victor and Yang 2012; Yang and Victor 2011). In addition, some studies found entirely different trends of loneliness across age, such as an inverted U-shape, which emphasizes the need for more longitudinal research (Mund et al. 2020; Schnittker 2007; Schultz and Moore 1988).

Theoretical frameworks like the social convoy model or socioemotional selectivity theory describe changes in social networks over the life course, postulating a decrease in network size with age (Carstensen 1992; Kahn and Antonucci 1980). The assumption is that younger individuals need a large social network to examine the scope of different values, aspirations, and opportunities to find out which path they want to take in life. Particularly, the social convoy model suggests the idea that adolescents and young adults may rely on a diverse set of social relationships for different types of support, including emotional, instrumental, and informational support. After this early life course stage, the choices a person makes become more targeted and consequently, stimulation from a large variety of social network members becomes less relevant. Instead, individuals begin to focus on fewer but close social network members. Hence, the socioemotional selectivity theory suggests that the motivation for social contact shifts from quantity to quality as individuals age.

A meta-analysis by Wrzus and colleagues (2013), encompassing both cross-sectional and longitudinal studies, found that the social network size expands until young adulthood before it gradually declines with increasing age. More specifically, they observed that the family network size remained stable from adolescence to old age, while the number of friends within individuals’ social networks decreased throughout adulthood. Furthermore, they detected a larger number of confidants and friends in social networks of student samples. This observation is important, as it indicates a larger social network size as well as more close social ties among younger individuals or at least a subgroup of younger individuals.

A longitudinal study by Sander and colleagues (2017) found that the face-to-face contact frequency with family members remains equally stable throughout the life course, whereas the frequency of face-to-face interactions with friends, neighbors, and acquaintances decreases after young adulthood. Shaw and colleagues (2007) conducted a 10-year longitudinal study among non-institutionalized individuals aged 65 years or older and found that the overall contact frequency, face-to-face and media-based contact, remained relatively stable for family members but declined substantially among friends. These results align with the concept of normative, age-related shifts in social networks postulated by the social convoy model and socioemotional selectivity theory.

Since the density of social networks and overall frequency of face-to-face interactions with social network members appears to be higher for younger individuals (Nicolaisen and Thorsen 2014; Sander et al. 2017; Wrzus et al. 2013), larger losses of contact due to the pandemic-induced restrictions should lead to a larger increase in loneliness among younger than middle-aged or older individuals. Findings from cross-sectional studies in the United Kingdom and Spain support the hypothesis of age-normative differences, since higher levels of loneliness were observed among younger individuals compared to older age groups during the lockdown (Bu et al. 2020; Losada-Baltar et al. 2021). Although it can be argued that younger individuals are more adapted and capable to substitute a lack of regular face-to-face interactions with media-based communication, previous studies suggest that the mode of social interactions plays a notable role when it comes to the prevalence of loneliness. Frequent face-to-face contact is associated with lower levels of loneliness, whereas frequent online social interactions are associated with higher levels of loneliness (Luhmann and Hawkley 2016), suggesting that media-based alternatives cannot substitute a lack of face-to-face interactions.

A fundamental challenge posed by COVID-19 restrictions was the hindrance of social network growth and maintenance. Social restrictions disrupted peripheral contact opportunities, such as those formed through school, university, or workplace interactions. Consequently, individuals faced difficulties in establishing new relationships and expanding their social networks, which is particularly important for younger individuals according to the socioemotional selectivity theory. Although media-based alternatives provided a means of staying in contact, a study by Reich and colleagues (2012) reveals an overlap between face-to-face and media-based interactions among adolescents. Their study indicates a preference for engaging with familiar social network members from offline contexts rather than predominantly forming new and unfamiliar contacts online.

It is essential to highlight that even during the most severe COVID-19 restrictions, Switzerland, the country our study is based on, permitted social gatherings of up to five individuals simultaneously. As a result, the qualitative aspect of social relationships was less heavily constrained since closeness and intimacy could be continuously experienced, whereas the quantitative aspect of social networks was profoundly restricted. Since the density of social networks and desired frequency of face-to-face interactions are generally higher for younger individuals (Sander et al. 2017; Wrzus et al. 2013), it appears evident that they faced more challenges in navigating compromises due to the implementation of social restrictions, increasing their likelihood to experience loneliness. Our first hypothesis is thus that younger individuals experienced a greater increase in loneliness due to the restrictions than middle-aged and older individuals.

### Loneliness by gender

The relationship between loneliness and gender has been widely researched, often without theoretical assumptions or a clear hypothesis and inconsistent empirical findings (Maes et al. 2019). While some articles show higher levels of loneliness in men, an increasing amount of literature indicates that women are more vulnerable to loneliness, and a tangible proportion do not find any gender differences (Luhmann and Hawkley 2016; Maes et al. 2019; Mahon et al. 2006; Pinquart and Sörensen 2003; Victor and Yang 2012). Empirical findings that study the prevalence of loneliness before and during the COVID-19 pandemic indicate a stronger increase in loneliness among women. Most of these observations are, however, based on cross-sectional studies, which means that the intraindividual change of loneliness could not be examined (Bu et al. 2020; Li and Wang 2020; Losada-Baltar et al. 2021).

Differences in the perceived impact of COVID-19 restrictions on loneliness are likely to occur due to a contrasting understanding and evaluation of loneliness. Men appear to emphasize the importance of a partner when they evaluate whether they are lonely more strongly than women (Dykstra and Fokkema 2007; Freak-Poli et al. 2022). They tend to have smaller support social networks as well (Borys and Perlman 1985; Dykstra and Fokkema 2007; Štípková 2021). Considering the impact that restrictive measures had on opportunities to participate in social gatherings, it seems plausible that women experienced a stronger increase in loneliness compared to men. After all, the restrictions in Switzerland did not prohibit meeting one’s partner, even if he or she lived abroad.

In addition to a greater increase in loneliness among women compared to men during the pandemic, an interaction effect between gender and age appears to exist. A longitudinal study found that during the pandemic women between the age of 18 to 29 years, and 60 years and above faced a higher increase in loneliness than men in the same age brackets (Wickens et al. 2021). A stronger increase for younger women was also observed in a cross-sectional study that did not find higher levels of loneliness for older women, however (McQuaid et al. 2021). Since restrictions limited the possibility to reach out to their social network to gather for face-to-face interactions, female adolescents, and young adults might be more affected than male ones due to their differing coping behavior in times of stress (McQuaid et al. 2021; Taylor et al. 2000; Wickens et al. 2021). As social networks tend to be the largest during young adulthood (Wrzus et al. 2013), young women could experience a stronger reinforcement of loneliness. On the other hand, older women could be more affected than men, because they are more likely to be widowed (Federal Statistical Office 2022). Hence, our second hypothesis assumes that loneliness increased more strongly for women than for men and that this increase was more profound for young and older women than middle-aged ones.

### Loneliness by living arrangements

Living arrangements affect loneliness because they shape individuals’ social interaction opportunities and feelings of belongingness (Beutel et al. 2017; Dahlberg et al. 2022). Considering that face-to-face social interactions with a common household member are easy to initiate, it appears evident that living alone would make individuals more prone to loneliness (Beutel et al. 2017; Sundström et al. 2009). We assume that the restrictions following the COVID-19 pandemic led to a higher increase in loneliness among individuals living alone since possibilities to socially interact outside of the household were hampered.

In line with our expectations, Canadians who lived alone during the COVID-19 pandemic reported higher levels of loneliness than those living with family members or their partner (McQuaid et al. 2021; Wickens et al. 2021). Similar findings have been observed for European countries (Arpino et al. 2022; Atzendorf and Gruber 2021; Bu et al. 2020).

However, living arrangements and age are interrelated (Federal Statistical Office 2022): children and adolescents tend to live together with their parents, young adults may still live with their family, in shared flats or sometimes alone, middle-aged adults are more likely to share their household with a partner and/or children, and those of advanced old age tend to live together with their partner or alone, if they do not reside in nursing homes. While living alone should generally increase loneliness levels during the pandemic, a more complex interdependency between age and household composition may exist, given that young individuals tend to be unable to choose with whom they live together compared to middle-aged and older ones. We expect that living together with a partner had a positive effect and reduced loneliness compared to single-person households for all ages, as it constitutes a voluntary decision and because relationships have a qualitative and meaningful influence on experienced loneliness (Victor and Yang 2012). Previous studies support the notion that living together with a partner is a key factor that is associated with a decreased risk of experiencing loneliness (Greenfield and Russell 2011; Kim and Fredriksen-Goldsen 2016).

Hence, our third hypothesis presumes that individuals who live in shared households experience a smaller increase in loneliness than individuals who live alone. A positive effect should be noticeable for individuals that live together with their partner regardless of age while living in a shared household itself should prevent an increase in loneliness more strongly for middle-aged and older individuals because young people are often unable to choose whom to live with.

### Loneliness by perceived health risk

Individuals at risk of experiencing complications if they contract a COVID-19 infection were more likely to comply with social distancing measures (Miguel et al. 2021; Xu and Cheng 2021). They might even voluntarily reduce the number of face-to-face interactions with social network members further than imposed by their respective government. For instance, diabetes patients displayed more adherence to hygiene and social distancing rules, such as the avoidance of public places and public transports (Musche et al. 2021). This made them more likely to experience a disproportional increase in loneliness.

Given that age correlates positively with multimorbidity (Marengoni et al. 2011; Van den Akker et al. 1998), it is possible that among those who perceived themselves to be at risk of complications, loneliness increased more strongly for older individuals. Hence, our fourth hypothesis postulates that individuals who consider themselves to be at risk of developing complications from a COVID-19 infection experience a stronger increase in loneliness. Furthermore, we suspect that an interaction between age and health risk perception intensifies the increase in loneliness among older individuals.

## Country context, data, and measures

### Country context

Restrictive measures during the first wave of the COVID-19 pandemic were moderate in Switzerland when compared to other European countries that enforced strict lockdowns (Hirsch 2020). Nevertheless, a variety of restrictions were imposed that inevitably limited the possibility to socially interact with others (Federal Office of Public Health 2022; The Federal Council 2020). In March 2020, except for grocery shops and pharmacies, most retailer and several public institutions were closed, such as schools, museums, libraries, stores, markets, restaurants, bars, and night clubs. Home office was recommended and partially made compulsory. It was prohibited to meet with more than 5 people at the same time. Between the end of April and beginning of May, some restrictions were lifted (Federal Office of Public Health 2022). However, social distancing measures remained, and the ban that limited private gatherings was effective until June 1. Other restrictions, such as the ban of public events, was eased on June 6.

### Data

We use data from the Swiss Household Panel (SHP), an annual panel survey that collects information about living conditions and social change (Tillmann et al. 2022, SHP Group 2022), based on a probability-based sample of the Swiss population living in private households. From May 12 to June 26, 2020, a between-wave SHP survey was carried out to assess the impact of the COVID-19 pandemic. Respondents of the previous annual panel wave, which was collected between September 2, 2019, and March 3, 2020, were invited to participate in the COVID-19 wave (Refle et al. 2020). A total of 5843 out of 8772 individuals responded, equaling a response rate of 67% (Voorpostel et al. 2020).^1^ Although response bias does not appear to be a large issue (Kuhn et al. 2021), we used weights to present nationally representative estimates for Switzerland. The sample size amounts to 5782 individuals who gave a valid response to our dependent variable in both waves.
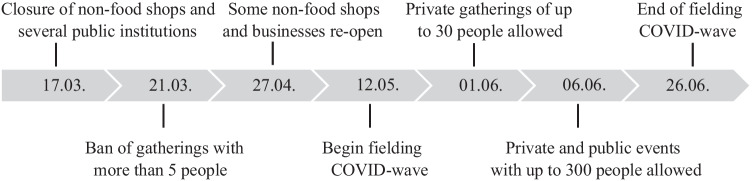


### Measures

Our dependent variable is *loneliness*. Participants were asked to answer the question “How lonely do you feel in your life, if 0 means ‘not at all lonely’ and 10 ‘extremely lonely’?” Before the COVID-19 pandemic, the average level of loneliness in the sample was 1.86, and it increased to 2.23 in the COVID-19 wave.

Our multivariate models used individual change scores to assess the impact of COVID-19 restrictions on loneliness. The change scores were obtained by subtracting the loneliness levels during the imposition of social restrictions from pre-pandemic levels. By employing change scores, we produce unbiased results if the parallel trend assumption holds, i.e., individuals with varying levels of loneliness would have experienced similar developmental patterns in the absence of the COVID-19 pandemic (Morgan and Winship 2015). We abstain from using lagged dependent variable models due to their tendency to yield inconsistent estimators (Brüderl and Ludwig 2015).

We used the following independent variables for our analysis: *age groups* (14–29, 30–64, 65–79 and 80–99 years old), *gender, living arrangements*, one assessing the household size (single- vs. multi-person household), and one measuring whether respondents lived together with their partner if they had one, as well as *health risk*. Whether or not respondents believed that they experience a heightened *health risk* in form of complications if they get infected with COVID-19 was measured through a simple "yes" or "no" question. Furthermore, we included a dummy variable to separate participants that answered before and after the measures were gradually eased on June 1 and accounted for differences in the data collection modes. The descriptive statistics of the variables that we used for the analysis are provided in Table 2 in the appendix. To reduce bias stemming from item non-response, we imputed a small number of missing values for our independent variables using chained equations (Azur et al. 2011).^2^ The employed imputation technique allowed us to generate plausible values for missing data points through iterative regressions based on observed values and enabled us to retain our sample size.

## Results

### Bivariate results

**Fig. 1:**
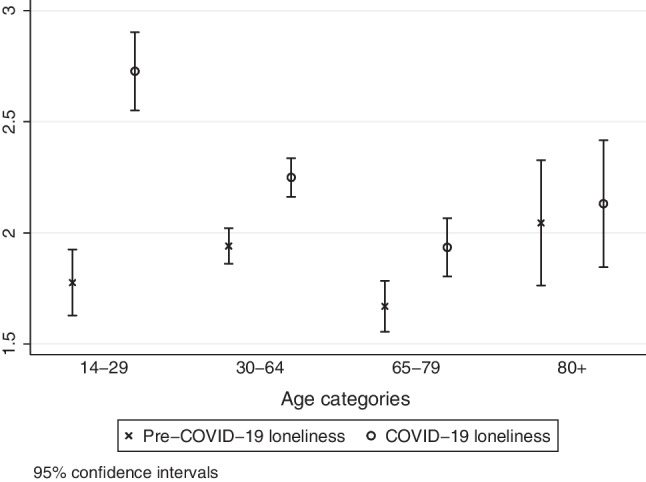
Mean level of loneliness by age category before and during COVID-19 restrictions. Data: SHP 2019/2020 and SHP COVID-19 Study 2020, weighted, (*N* = 5782)

Figure 1 shows the loneliness levels before and during the time of restrictions by age groups. Individuals between 14 and 29 years experienced, on average, an increase in loneliness by 1 point, followed by those aged 30–79 years old, who experienced an increase in loneliness that was about 0.2 points. The ones aged 80 years or older experienced no tangible increase.

Although the age categories that we distinguished are based on the theoretical context of life course differences, the cutoff points remain nonetheless to some extent arbitrary. We therefore examined the change of loneliness by using age as a continuous variable to control the robustness of our findings, using lowess smoothed plots (see Fig. 3 in the appendix). Due to the limited number of participants above the age of 85 years, we included individuals younger than 86 years for this visualization. The lowess plots reproduced the results of Fig. 1.

**Fig. 2:**
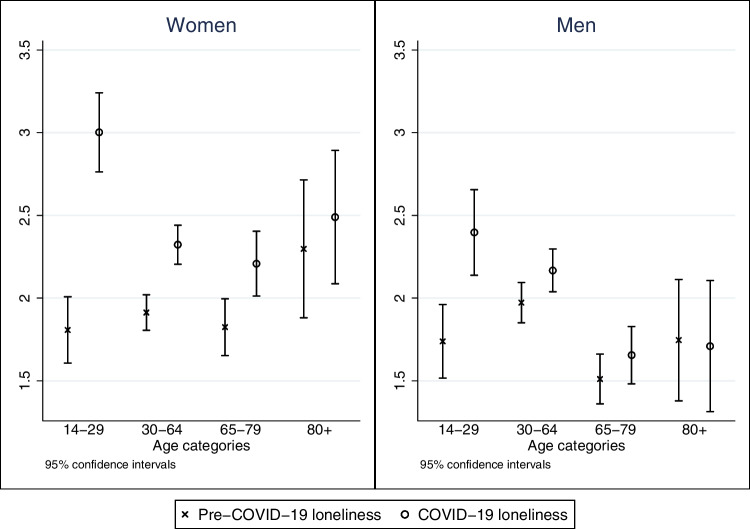
Level of loneliness before and during COVID-19 restrictions for women and men. Data: SHP 2019/2020 and SHP COVID-19 Study 2020, weighted (*N* = 5782)

**Fig. 3:**
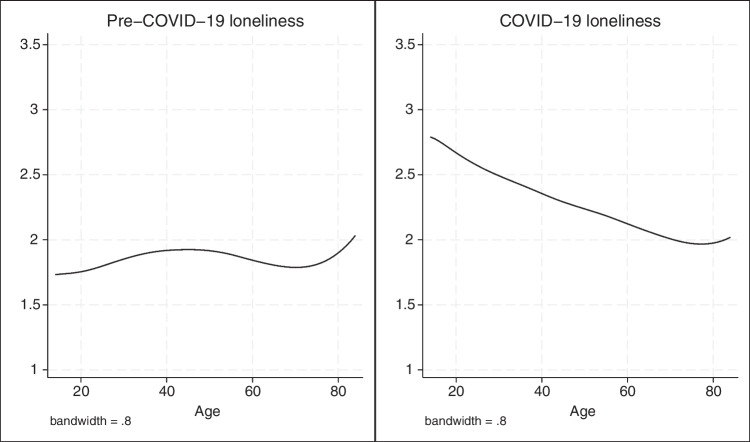
Mean loneliness by age treated as a continuous variable before and during COVID-19 restrictions. Data: SHP 2019/2020 and SHP COVID-19 Study 2020, weighted and lowess smoothed, respondents up to 85 years (*N* = 5695)

Figure 2 shows differences in changes of loneliness by age groups separately for women and men. Starting from comparable loneliness levels, the increase during the pandemic was steeper for females between 14 and 29 years of age. Although women experienced a larger increase in loneliness across all age groups, the difference was smaller for the remaining three age groups. Interestingly, men aged 80 years and older reported a constant level of loneliness. It is noticeable that women above the age of 64 years reported higher levels of loneliness on average than equally old men before the pandemic, which means that those women were lonelier to begin with and experienced a stronger increase in loneliness during the implementation of restrictions as well.

### Multivariate results

To control for confounding factors and to analyze interaction effects between age and our independent variables, we estimate multivariate linear regression models.

We include two control variables in all models: the response date in the COVID-19 wave for the time of fielding, and the data collection modes in the pre-COVID-19 wave (telephone vs. web) and in the COVID-19 wave (web in vs. paper and pen). The positive but insignificant coefficient of the response date means that there is no decrease of loneliness despite the possibility to meet an increased number of people face-to-face again with the beginning of June 2020.^3^

The first model M1 includes age categories and shows, consistent with our previous findings, that loneliness increased the most for the youngest age group (see Table 1). The changes in loneliness levels between the youngest and all other age categories are statistically significant, while the changes are not statistically significant across the three older age categories.

**Table 1:** Change in loneliness levels due to the implementation of COVID-19 restrictions

	M1	M2	M3	M4
Age (ref. 14–29)				
30–64	− 0.692***	− 0.742***	− 0.525***	0.275
65–79	− 0.719***	− 0.910***	− 0.678***	0.221
80–99	− 0.841***	− 1.114***	− 0.896***	− 0.246
Female		0.278***	0.265***	0.265***
Health risk		0.272**	0.268**	0.274**
Shared Household		− 0.276**	0.0951	0.870**
Living with partner			− 0.458***	− 0.392***
30–64 × Shared Household				− 0.876*
65–79 × Shared Household				− 1.009**
80–99 × Shared Household				− 0.609
From June 1st	0.148	0.208^+^	0.195^+^	0.203^+^
Telephone in pre-Covid-19 wave	1.282***	1.247***	1.251***	1.247***
Web in Covid-19 wave	0.376**	0.463***	0.464***	0.468***
Constant	− 0.531*	− 0.501*	− 0.710**	− 1.442***
*R*^2^	0.022	0.028	0.031	0.032
AIC	27454	27423	27407	27406
BIC	27501	27490	27481	27499

Source: SHP 2019/2020 and SHP COVID-19 Study 2020, weighted (*N* = 5782)

Change score models, ^+^*p* < 0.10, * *p* < 0.05, **p < 0.01, ***p < 0.001

Since the variables in model M3 fit the data better than model M2 (LR chi2(3) = 17.0; Prob > chi^2^ = 0.01) and model M4 better than model M3 (LR chi2(3) = 7.8; Prob > chi^2^ = 0.05), we discuss implications of the models M3 and M4 in depth. Model M3 includes all independent variables but no interaction and depicts a strong divergence between age categories reflecting our bivariate results (see Fig. 1). In line with our previous results, women experienced a stronger increase in loneliness than men and individuals who believe they experience a heightened health risk became lonelier than those who do not expect complications. Sharing a household or living with a partner reduces the increase in loneliness when both variables are included separately. When they are included in the same model, however, those living in shared households do not show a statistically significant difference in their evolution of loneliness anymore (see M3).

Once we include the interaction effect between household size and age in model M4, we do no longer observe any differences between age categories in the main terms. These age main effects must be interpreted as effects that compare individuals in single households. It is evident that no statistically significant difference between age groups exists for single-person households. However, the interaction shows that 14–29-year-olds experienced a significant increase in loneliness compared to individuals aged 30–79 years among those that lived in shared households. This means that the youngest did not benefit from living in shared households during the time of restrictions. Although the coefficient suggests that a shared household protects against an increase in loneliness for individuals aged 80 years or above as well, the interaction between household size and individuals of advanced old age does not reach statistical significance because of the small group size in our sample.

We tested three more interactions in model M3 but observed no interdependency between age and gender, age and perceived health risks, or age and living with a partner. This means that the main effects of these variables are independent and do not interact with age groups.

Since the measurement of loneliness is likely to be affected by measurement error, we tested the same models using a dependent variable based on the reliable change index, similarly, to van der Velden and colleagues (2021). While the reliable change index is widely used to account for measurement error when evaluating change scores, it is not a panacea (McAleavey 2022). The reliable change index is often used to evaluate the reliability of a difference score between two observations from the same individual. It is defined as

$${\text{RC}} = \frac{{x_{2} - x_{1} }}{{\sqrt {2\left( {s_{1} \sqrt {1 - r_{xx} } } \right)^{2} } }},$$

with *x*_1_ and *x*_2_ denoting the level of loneliness at time 1 and 2, *r*_xx_ the reliability (Cronbach’s alpha) and *s*_1_ the standard deviation of *x*_1_. Dividing the raw change score by the “standard error of the difference score” ensures that measurement errors are taken into account; however, it is important to note critiques highlighted by McAleavey (2022). Next, we define a variable, collapsing the RC into three groups, depending on the “clinically significant change” of the raw change score: those who are less lonely during Covid-19 (RC < − 1.96; (variable set to − 1), those with an unchanged loneliness score (− 1.96 <  = RC <  = 1.96; (variable set to 0) and those who are lonelier during Covid-19 (RC > 1.96; (variable set to 1).

Out of 5782 respondents, 274 reported reduced loneliness during the Covid-19 pandemic, 4965 indicated no change, and 543 an increase in loneliness. The limited instances of significant changes in loneliness levels underscore the conservative nature of the reliable change index.

To estimate our models, we used ordered logit models. The results support the findings from the regressions that used raw change scores, although the coefficients in model M3 for individuals aged 30–64 years are no longer significantly different from those aged 80–99 years.

## Discussion and conclusion

The implementation of COVID-19 restrictions led to a disproportionate increase in loneliness among different age groups. While several studies emphasized that the overall level of loneliness increased, our study provides a more detailed analysis by examining intraindividual changes shortly before the onset of the pandemic and during the first lockdown in spring 2020. We used panel data from the SHP to assess the magnitude of the effect that restrictions had on individuals’ changed loneliness levels and uncovered four main findings.

First, the mean change of loneliness was higher among the youngest compared to middle-aged, older, and individuals of advanced old age. This finding is in line with our hypothesis, suggesting that younger individuals faced greater challenges in compromising and fulfilling their desire for face-to-face interactions with their comparatively larger social networks during the COVID-19 restrictions. The notion that the density of social networks is higher for younger individuals corresponds to the theoretical assumptions of the social convoy model and socioemotional selectivity theory (Carstensen 1992; Kahn and Antonucci 1980). Both postulate a decline in social network sizes after young adulthood. This predicament was likely compounded by younger individuals’ inclination of higher contact frequencies with their social network members, increasing their susceptibility to loneliness. Although longitudinal research shows that the overall contact frequency remains stable regarding family members throughout the life course, the contact frequency with friends diminishes over time. According to the social convoy model and socioemotional selectivity theory, quantitative aspects of social networks are particularly relevant for adolescents and young adults, who still need to broaden and develop their values, aspirations, and personalities in order to make important life choices. What resonates with this theoretical framework are not only behavioral but also age-normative patterns of expectations. Consistent with our hypothesis and results, Victor and Yang (2012) show that the frequency of social contact is a better indicator for loneliness among younger individuals, whereas the quality of social relationships is more strongly associated with loneliness among middle-aged and older individuals. Since face-to-face encounters were not entirely prohibited, but temporarily limited to a maximum of five people per gathering during COVID-19 restrictions, it appears plausible that particularly those above the age of 65 years experienced the consequences of the restrictions as less severe, since they would have been capable to continuously meet with few but close social network members.

Second, while loneliness increased more strongly for women than for men, we did not observe an interdependency between age and gender. The first finding is in line with the fact that men emphasize the importance of having a partner when it comes to factors that protect against loneliness (Dykstra and Fokkema 2007). Since face-to-face contact between partners was not impeded due to the COVID-19 pandemic and women tend to rely more on their support network in times of stress, it appears plausible that they experienced a stronger increase. However, young women were not more strongly affected than middle-aged or older ones.

Third, individuals who lived in shared households or together with their respective partner during the time of COVID-19 restrictions experienced a lower increase in loneliness. Although we did not find interaction effects between age and living together with a partner, our results depict an interdependency between age and shared households. Those who lived in single-person households experienced an increase in loneliness during the pandemic, independent of their age. But individuals below the age of 30 years did not benefit from living in shared households, whereas those aged 30 to 79 years benefitted considerably from sharing their household with at least one other person. Hence, while living together with a partner appears to protect against loneliness regardless of age, the composition of multi-person households plays a role when it comes to the prevention of increased loneliness. A possible explanation for this finding is the voluntary nature of household compositions: Middle-aged and older adults usually make a conscious decision whether to live with other people, whereas adolescents and young adults are more likely to lack the independency to choose. Whereas adolescents could possibly feel pressured to further limit their face-to-face meetings to protect their parents from infections, young adults might be stuck within shared flats with roommates that they do not feel close with.

Forth, those who considered themselves to be at risk of experiencing complications if they contracted a COVID-19 infection, became lonelier than those who did not. This effect was not amplified by belonging to an older age group. Given that increased age correlates positively with health-related issues (Marengoni et al. 2011; Van den Akker et al. 1998), we expected an interaction that would increase loneliness for older individuals more strongly among the people that considered themselves to be at risk. No such interdependency was found. Since previous research results found that individuals adapted their social interactions and behavior when they assumed they were exposed to a greater health risk in case of a COVID-19 infection (Musche et al. 2021), it seems that the phenomenon is a general one.

The longitudinal design of our study allowed us to examine the evolution of loneliness levels before and during the implementation of restrictions. We presented the intraindividual change of individuals, which reduces bias caused by omitted variables. However, a technical limitation affects our study: The fielding of the COVID wave covered the time of strongest restrictions but also a period of eased restrictions, although we observed no significant difference related to changes in loneliness levels between those two fielding periods. Another limitation of this study is that changes in loneliness were analyzed based on a unidimensional measure comprising a single item. The Swiss Household Panel does not provide more comprehensive measures of loneliness, such as the De Jong Gierveld Loneliness Scale (Gierveld and Tilburg 2006) or SELSA-S (DiTommaso et al. 2004), which could have enabled a finer distinction between the emotional and social dimensions of loneliness.

Despite these limitations, age differences clearly suggest that loneliness increased disproportionately due to COVID-19 restrictions. Older individuals were less negatively affected than younger ones, which appears to be partially explainable through household compositions and living arrangements that differ according to life course stages. Furthermore, we can conclude that loneliness does not appear to drop immediately after restrictions are lifted, raising the question of whether long-term effects may occur, or if loneliness eventually returns to pre-pandemic levels after a few months. Further exploration in this area would be valuable for a more comprehensive understanding of the impact of COVID-19 restrictions on loneliness over time.

## Data Availability

This study used data collected by the Swiss Household Panel, which is based on the Swiss Centre of Expertise in the Social Sciences (FORS).

## Appendix

See Fig. 3 and Table 2.

**Table 2:** Descriptive statistics

Variables	Mean	SD
*Loneliness*		
Before COVID-19	1.77	2.23
During COVID-19	2.18	2.51
*Time of fielding*		
Before June 1st	0.66	0.47
From June 1st	0.34	0.47
*Mode Pre-COVID-19 wave*		
Phone (vs. web)	0.95	0.22
*Mode COVID-19 wave*		
Web (vs. paper and pen)	0.67	0.47
*Age*		
14–29	0.14	0.34
30–64	0.55	0.50
64–79	0.26	0.44
80–99	0.06	0.23
*Gender*		
Female (vs. male)	0.55	0.50
*Living arrangements*		
Single household (vs. shared)	0.19	0.39
Living without partner (vs. with partner)	0.32	0.47
*Health*		
Not at risk (vs. at risk)	0.66	0.48

SHP 2019/2020 and SHP COVID-19 Study 2020, unweighted (*N* = 5782)

## Footnotes

1 The data collection mode differed between the waves. Ninety-five percentage of the pre-COVID-19 wave was assessed through telephone interviews and 5% through web-interviews. Regarding the COVID-19 wave, 67% responded via web-interviews and 33% through pen and paper interviews. Since the modes may have influenced the answers (Klausch et al. 2013), we control the mode of data collection before (phone vs. web) and during (paper vs. web) the COVID-19 wave in our multivariate models.

2 6 for household size, 64 for living with partner and 364 for perceived health risk.

3 This could also be a late case effect, since later respondents are often less motivated than earlier ones (Kreuter et al. 2010) and motivation is likely to be correlated with variables like loneliness. Similarly, responding by telephone in the pre-COVID-19 wave and by web in the COVID-19 wave may be early case effects, since the complementary modes (web in the pre-COVID-19 wave and paper and pen in the COVID-19 wave) are follow-up modes. In sum, the relatively strong effects of telephone (pre-COVID-19 wave) or web (COVID-19 wave) in Table 1 may be true mode effects and/or sample selection effects.

## Code availability

Stata 18 SE was used for the statistical analysis.

## Data deposition information

This study used data from wave 21, collected between 2019 and 2020, and the COVID-19 wave of the Swiss Household Panel available at https://www.swissubase.ch/en/catalogue/studies/6097/19347/datasets.
